# Association between opioid prescription profiles and adverse health outcomes in opioid users referred for sleep disorder assessment: a secondary analysis of health administrative data

**DOI:** 10.3389/frsle.2023.1161857

**Published:** 2023-07-07

**Authors:** Tetyana Kendzerska, Atul Malhotra, Andrea S. Gershon, Marcus Povitz, Daniel I. McIsaac, Shawn D. Aaron, Gregory L. Bryson, Robert Talarico, Michael Godbout, Peter Tanuseputro, Frances Chung

**Affiliations:** ^1^The Ottawa Hospital Research Institute, The Ottawa Hospital, Ottawa, ON, Canada; ^2^Faculty of Medicine, Department of Medicine, University of Ottawa, Ottawa, ON, Canada; ^3^ICES, Ottawa/Toronto, ON, Canada; ^4^UC San Diego, San Diego, CA, United States; ^5^Department of Medicine, University of Toronto, Toronto, ON, Canada; ^6^Department of Medicine, Sunnnybrook Health Sciences Centre, Toronto, ON, Canada; ^7^Department of Medicine, Cumming School of Medicine, University of Calgary, Calgary, AB, Canada; ^8^Departments of Anesthesiology and Pain Medicine, University of Ottawa and The Ottawa Hospital, Ottawa, ON, Canada; ^9^Bruyère Research Institute, Ottawa, ON, Canada; ^10^Department of Anesthesia and Pain Medicine, University Health Network, University of Toronto, Toronto, ON, Canada

**Keywords:** opioids, adverse health outcomes, sleep assessment, mortality, inpatient visits, opioid poisoning

## Abstract

**Background:**

Information is needed to guide safe opioid prescribing in adults referred for a sleep disorder assessment. Previous studies have shown that individuals referred for a sleep disorder assessment have a higher likelihood of long-acting opioids and higher opioid dosages prescription than the general population, suggesting that these individuals are more at risk for opioid-related adverse health consequences.

**Methods:**

We included all adults who underwent a diagnostic sleep study (index date) in Ontario, Canada, between 2013 and 2016 (*n* = 300,663) and filled an opioid prescription overlapping the index date (*n* = 15,713). Through provincial health administrative databases, individuals were followed over time to assess the association between opioid use characteristics and 1-year all-cause mortality, hospitalizations and emergency department (ED) visits, and opioid-related hospitalizations and ED visits within extended follow-up to 2018.

**Results:**

Controlling for covariates, chronic opioid use (vs. not) was significantly associated with increased hazards of all-cause mortality [adjusted hazard ratio(aHR): 1.84; 95% confidence interval (CI): 1.12–3.02], hospitalization (aHR: 1.14; 95% CI: 1.02–1.28) and ED visit (aHR: 1.09; 95% CI: 1.01–1.17). A higher opioid dosage [morphine equivalent daily dose (MED) >90 vs. ≤ 90 mg/day] was significantly associated with increased hazards of all-cause or opioid-related hospitalization (aHR: 1.13; 95% CI: 1.02–1.26 and aHR: 2.27; 95% CI: 1.53–3.37, respectively). Morphine or hydromorphone prescription (vs. oxycodone) was significantly associated with an increased hazard of all-cause hospitalization (aHR: 1.30; 1.07–1.59 and aHR: 1.43; 95% CI: 1.20–1.70, respectively). Hydromorphone or fentanyl prescription (vs. oxycodone) was significantly associated with an increased hazard of opioid-related ED visit and/or hospitalization (aHR: 2.28, 95% CI: 1.16–4.47 and aHR: 2.47, 95% CI: 1.16–5.26, respectively).

**Conclusion:**

Findings from this retrospective study may inform the safe prescribing of opioids in adults referred for a sleep disorder assessment.

## Introduction

Historically high rates of prescription opioid use across North America (Gomes et al., 2014) led to an increased frequency of adverse opioid-related outcomes, such as mortality, hospitalizations, and emergency department (ED) visits (Gomes, 2017; Alsabbagh et al., 2021). Though the current epidemic is less driven by prescribed opioids (Gomes et al., 2018; Lovegrove et al., 2019), safe opioid prescribing is still important (Kurteva et al., 2021). For example, ~3% of previously opioid-naive individuals continued to use opioids for more than 90 days after major elective surgery (Clarke et al., 2014). Non-fatal opioid-related outcomes, such as hospitalizations or ED visits, have been reported, even when opioids were used as directed (Frood and Paltser, 2019; Rosen et al., 2019; Eckert and Yaggi, 2022). There is also a growing recognition of variations in effectiveness and safety between opioids due to differences in their pharmacokinetics and dynamics (Drewes et al., 2013). However, clinical trials are not designed to assess the long-term harms (Nury et al., 2022).

Literature suggested that 36–85% of people treated with prescription opioids may have sleep disordered breathing (SDB) (Lee-Iannotti and Parish, 2014; Chung et al., 2019; Mubashir et al., 2020). SDB is the most prevalent sleep disorder (Benjafield et al., 2019) and the most common reason for referral for sleep assessment. This high prevalence is concerning due to plausible mechanisms suggesting that opioids may alter sleep architecture and adversely impact respiratory function (Busse et al., 2017; Chung et al., 2019; Rosen et al., 2019) through a decrease in airway muscle tone, the output of the respiratory pacemaker, and central respiratory drive (Pattinson, 2008).

Previously, we found that adults referred for a sleep disorder assessment who used prescription opioids had a significantly increased hazard of all-cause mortality, hospitalizations and ED visits compared to non-opioid users, regardless of SDB presence (Kendzerska et al., 2022). We also demonstrated a higher prevalence of opioid use with a large proportion on long-acting opioids and higher opioid dosages, and a higher use of benzodiazepines among adults referred for a sleep disorder assessment than the general population (Kendzerska et al., 2020), suggesting that these individuals are more at risk for opioid-related adverse health consequences. However, there is a need to further explore specific opioid characteristics associated with the greatest risk for both general and opioid-related adverse outcomes in opioid users referred for sleep disorder assessment (Kendzerska et al., 2022). Although the best therapeutic option for opioid-related adverse health outcomes may be the withdrawal of opioids, health providers, especially primary care physicians, are often faced with the challenge of effectively managing the underlying disorder while ensuring individual safety, which requires evidence of a safe prescribing of opioids in this population.

To address this knowledge gap, utilizing provincial health administrative databases, we assessed the characteristics of opioids associated with an increased hazard of all-cause mortality (primary outcome) and all-cause or opioid-related hospitalizations and ED visits (secondary outcomes) in adults undergoing a diagnostic sleep study while being treated with prescription opioids. We hypothesized that among opioid users, chronic opioid use and a higher opioid dosage are associated with adverse health outcomes.

## Methods

### Study design

We conducted a retrospective longitudinal population-based cohort study utilizing provincial health administrative data (Ontario, Canada). The use of data in this project was authorized under section 45 of Ontario's Personal Health Information Protection Act, which is exempt from review by a Research Ethics Board.

### Data sources

ICES is a non-profit independent research institute whose legal status under Ontario's health information privacy law allows it to collect and analyze health care and demographic data, without consent, for health system evaluation and improvement. Since 1991, high-quality individual-level administrative databases (Juurlink et al., 2006) on publicly funded services for all Ontario residents provided by hospitals and physicians and are housed at ICES, including information on outpatient and outpatient visits and procedures, such as sleep studies (ICES, 2005), and information on dispensed prescriptions for controlled substances such as narcotics and benzodiazepines. We used the following databases in this study: the Registered Persons Database (RPDB); the Same Day Surgery (SDS) and National Ambulatory Care Registry System (NACRS) databases; the Ontario Health Insurance Plan (OHIP) database; the Canadian Census; ICES-derived disease-specific databases; the Narcotics Monitoring System (NMS) and the Assistive Devices Program (ADP) databases. Details on all databases used are provided in the Supplementary material 1 and at https://datadictionary.ices.on.ca/Applications/DataDictionary/. These datasets were linked using unique encoded identifiers.

### Study population

Our population was drawn from an existing dataset of all adults 18 years and older who underwent a diagnostic sleep study (***index date***) between ***July 1, 2013, and June 30, 2016***. Given the lack of information on SDB severity and other sleep disorders in health administrative data, we assumed that those individuals referred for a sleep disorder assessment were at higher risk for sleep disorders than the general population (Kendzerska et al., 2021). Only those individuals who filled an opioid prescription between July 2012 and June 2016, with a days-supply overlapping the index date, were included. We chose this time to ensure (i) a 1-year lookback to identify opioid users and opioid characteristics through the NMS, given its availability since 2012, and (ii) at least a year of follow-up to capture outcomes of interest. Everyone was followed from the index date until the end of the study (March 31, 2018), emigration from Ontario, or until death, whichever came first.

We excluded individuals who were: (1) in long-term care (Tanuseputro et al., 2017b) or received palliative care (Tanuseputro et al., 2017a) in the year prior to the index date; (2) previously treated for SDB; or (3) taking opioids that are rarely used and/or with no well-defined morphine equivalencies such as intranasal, injectable, or rectal suppositories. Details on inclusion and exclusion criteria are also provided in the Supplementary Table 1.

### Exposures: opioid-related characteristics

All opioids dispensed during the study period were identified through the NMS database, including oral formulations of morphine, codeine, oxycodone, hydromorphone, meperidine, pentazocine, tramadol, tapentadol, opium, as well as transdermal fentanyl and buprenorphine patches, and opioid maintenance therapy (OMT), that includes buprenorphine for opioid dependence (Subutex), buprenorphine/naloxone, and methadone for opioid dependence. Opioid exposures were followed for a minimum of 1 year following the index date to identify the opioid prescription pattern in follow-up.

At the index date, we considered the following characteristics: (Webster et al., 2008; Van Ryswyk and Antic, 2016) (i) chronic opioid use (three or more prescriptions for any opioids in the last 6 months or at least one prescription for a long-acting opioid) (Kendzerska et al., 2020), (ii) opioid daily dose (morphine equivalent daily dose, MED), (iii) specific opioid types, (iv) being on opioid maintenance therapy (Nguyen et al., 2016), and (vi) being on more than one opioid medication at the index date. For an individual on more than one opioid at the index date, the medication responsible for the maximum days' supply was chosen to define the specific opioid type at the index date.

At the individual level, we calculated the total, mean and maximum MED on the index date based on the number of tablets dispensed, the strength of the medication, and the number of days' supply. MED was considered as both a continuous variable and categorical variable (< 90 vs. ≥90 mg/day). (Busse et al., 2017; Kurteva et al., 2021) The threshold of 90 mg was selected because the CDC Guideline for Prescribing Opioids for Chronic Pain suggested that opioid dosages should not be increased to ≥90 MED “without careful justification based on diagnosis and on individualized assessment of benefits and risks” (Dowell et al., 2016).

In explanatory analysis, we examined the opioid prescription pattern within 180 days since the index date. While comparing MED at the index date to the MED at the first prescription that occurred within 180 days of the index date, individuals were classified as “dose reduction,” “dose increase,” or “the same dose as at the first refill” groups (Shah et al., 2017). Those with no opioid prescription within 180 days of the index date were classified as the “No refill” group.

### Outcomes

Our primary outcome was all-cause mortality *within the first year since the index date*. As secondary outcomes, we considered all-cause ED visits and hospital admissions *within the first year since the index date*. One-year follow-up was chosen for general outcomes to ensure relevance to the opioid prescription overlapping the index date. We also considered as a secondary outcome hospitalizations or ED visits due to opioid poisoning (Fernandes et al., 2016; Goverment of Canada, 2021). Given a small sample size for the opioid-related outcome within the first year since the index date, we extended the follow-up for this outcome until the end of the study (March 31, 2018) to increase statistical power.

### Confounders and risk factors

The following factors were considered: (i) demographics at the index date: age, sex, place of residence, neighborhood income quintile as a measure of socioeconomic status; (Pampalon et al., 2009) prior to the index date: (ii) prescription of benzodiazepines in the last year; (FDA, 2016) (iii) prevalent comorbidities (diabetes, hypertension, mental health conditions, arthritis, asthma, COPD, cancer, cardiovascular, liver and kidney diseases); (iv) Charlson comorbidity index; (Deyo et al., 1992) (v) any outpatient or inpatient surgical intervention in the last year; (vi) any hospitalization or ED visits in the past year; and (vii) substance use (Gomes et al., 2017; Webster, 2017) (including opioid use) disorder and neuromuscular disorder in the last 5 years. SDB-related treatment (positive airway pressure therapy or surgical interventions) in follow-up was considered as a time-varying covariate since the index date. Details on definitions are provided in the Supplementary Table 1 and our previous work (Kendzerska et al., 2020, 2022).

### Analyses

Descriptive statistics were used to characterize the study population. We calculated standardized differences to compare baseline characteristics.

We used multivariable Cox proportional hazards regressions to assess associations between opioid characteristics and each outcome, controlling for the covariates described above. ***For the primary analysis, only individuals with oral administration of opioids were*** ***considered in the regression model. We also excluded individuals on OMT*** as those individuals may represent a very different population. When comparing the specific type of opioids, all statistical models were additionally adjusted for MED at the index date; *oxycodone* was considered a reference group, given that according to the Canadian Guideline for Safe and Effective Use of Opioids for Chronic Non-cancer Pain, morphine, oxycodone or hydromorphone are recommended as a first-line treatment for severe pain, with fentanyl being a second line (McMaster University, 2010; Kahan et al., 2011a).

In the secondary analyses, due to potential serious risks and death when combining opioids with benzodiazepines (FDA, 2016), we assessed if a prescription for benzodiazepines in the year prior to the sleep study modifies the relationship between opioid characteristics and outcomes through statistical interactions. We also investigated the association between the OMT and the outcomes of interest, controlling for confounders. Finally, we conducted an analysis to explore the relationship between outcomes and opioid dosage change at the first refill and opioid discontinuation within 180 days of the index date.

All statistical analyses were performed in the secure environment at ICES following Ontario privacy standards using SAS 9.3 (SAS Institute Inc., Cary, NC).

## Results

### Description of population and opioid characteristics

Of 300,663 adults who underwent a diagnostic sleep study between 2013 and 2016, 15,713 (5.2%) filled a prescription for opioids with a days-supply overlapping the date of the sleep study. The median age was 54 yrs., 7,689 (48.9%) were men, 4,283 (27.3%) resided in the lowest income quintile, 2,444 (15.6%) in rural areas, and 6,153 (39.2%) were prescribed benzodiazepines (Table 1).

**Table 1:** Population characteristics for the entire cohort of all adults who underwent a diagnostic sleep study between 2013 and 2016 while being treated with prescription opioids by the primary outcome, all-cause mortality within the first year since the diagnostic sleep study.

**Characteristics**		**Total**	**Died from any cause within the first year since the diagnostic sleep study**		**Standardized difference**
		***N*** = **15,713**	**No (*****N*** = **15,508)**	**Yes (*****N*** = **205)**	
**Demographics at the index date (the date of the diagnostic sleep study)**					
Age, years; median (IQR)		54 (46–63)	54 (46–63)	63 (55–74)	0.70
Sex: male		7,689 (48.9)	7,576 (48.9)	113 (55.1)	0.13
Neighbourhood income quintile (Q)	Q1 (lowest)	4,283 (27.3)	4,208 (27.1)	75 (36.6)	0.20
	Q2	3,574 (22.7)	3,532 (22.8)	42 (20.5)	0.06
	Q3	3,059 (19.5)	3,021 (19.5)	38 (18.5)	0.02
	Q4	2,606 (16.6)	2,582 (16.6)	24 (11.7)	0.14
	Q5 (highest)	2,156 (13.7)	2,130 (13.7)	26 (12.7)	0.03
Rurality: Yes		2,444 (15.6)	2,402 (15.5)	42 (20.5)	0.13
**Comorbidities, primary health care exposure, surgical interventions and controlled substance use**					
**in the last year prior to the sleep study**					
Charlson comorbidity index (CCI)	None (CCI = 0)	13,680 (87.1)	13,570 (87.5)	110 (53.7)	0.80
	Low (CCI = 1)	869 (5.5)	845 (5.4)	24 (11.7)	0.22
	Moderate (CCI = 2)	603 (3.8)	580 (3.7)	23 (11.2)	0.29
	High (CCI ≥ 3)	561 (3.6)	513 (3.3)	48 (23.4)	0.62
Number of primary care visits, median (IQR)		8 (5–14)	8 (5–14)	10 (5–15)	0.13
Surgery/intervention indicator		1,296 (8.2)	1,272 (8.2)	24 (11.7)	
Hospitalization/ED visits, median (IQR)		1 (0–2)	1 (0–2)	2 (0–4)	0.50
Benzodiazepine dispensed		6,153 (39.2)	6,061 (39.1)	92 (44.9)	0.12
Medical cannabinoids dispensed		664 (4.2)	656 (4.2)	8 (3.9)	0.02
Stimulants dispensed		383 (2.4)	378 (2.4)	≤ 5 (2.4)	0
**Comorbidities defined in the last 5 years prior to the sleep study**					
Alcohol dependence/intoxication		842 (5.4)	818 (5.3)	24 (11.7)	0.23
Neuromuscular disease		1,656 (10.5)	1,627 (10.5)	29 (14.1)	0.11
**Prevalent comorbidities**					
Chronic heart failure		1,241 (7.9)	1,176 (7.6)	65 (31.7)	0.64
Chronic obstructive pulmonary disease		4,383 (27.9)	4,267 (27.5)	116 (56.6)	0.62
Asthma		4,586 (29.2)	4,513 (29.1)	73 (35.6)	0.14
Coronary artery disease		2,753 (17.5)	2,680 (17.3)	73 (35.6)	0.42
Diabetes		4,723 (30.1)	4,620 (29.8)	103 (50.2)	0.43
Hypertension		8,453 (53.8)	8,298 (53.5)	155 (75.6)	0.47
Non-psychotic mood and anxiety disorders		6,480 (41.2)	6,406 (41.3)	74 (36.1)	0.11
Rheumatoid Arthritis		640 (4.1)	630 (4.1)	10 (4.9)	0.04
Cancer		1,205 (7.7)	1,165 (7.5)	40 (19.5)	0.36
**Prior opioid use disorder**					
Any opioid use disorder indication^*^		1,655 (10.5)	1,635 (10.5)	20 (9.8)	0.03
**Follow-up-related variables**					
SDB-related treatment initiated within the first year since the diagnostic sleep study		6,808 (43.3)	6,763 (43.6)	45 (22.0)	0.47
**Secondary outcomes**					
All-cause ED visits within the first year since the diagnostic sleep study		6,887 (43.8)	6,763 (43.6)	124 (60.5)	
All-cause hospitalization within the first year since the diagnostic sleep study		3,077 (19.6)	2,937 (18.9)	140 (68.3)	
Opioid poisoning hospitalization/ED visit		213 (1.4)	208 (1.3)	≤ 5 (2.4)	

Estimates presented as *n* (%) unless specified.

^*^Any opioid use disorder indication: hospitalizations/ED visits for opioid use disorder or mental and behavioural disorders due to the use of opioids 5-years prior to the index date or taking narcotics for opioid use disorder 1-year prior to the index date.

CCI, Charlson Comorbidity Index; ED, emergency department; IQR, interquartile range.

Details on the opioid types are presented in Table 2: 4,606 (29.3%) individuals were on more than one opioid at the index date, 13,098 (83.4%) were chronic opioid users, and 3,829 (24.4%) on MED > 90 mg/day. The most frequently single-agent opioids prescribed were hydromorphone (11.5%), oxycodone (6.7%), and morphine (6.1%).

**Table 2:** Overall opioid characteristics and by the primary outcome, all-cause mortality, within the first year since the diagnostic sleep study.

**Characteristics**	**Total**	**Died from any cause within the first year since the diagnostic sleep study**	
	***N*** = **15,713**	**No (*****N*** = **15,508)**	**Yes (*****N*** = **205)**
**At the index date**			
Being on more than one opioid	4,606 (29.3)	4,538 (29.3)	68 (33.2)
Chronic opioid use	13,098 (83.4)	12,913 (83.3)	185 (90.2)
**Formulation**			
Long-acting opioid dispensed	5,699 (36.3)	5,617 (36.2)	82 (40.0)
**Dosage**			
Total daily MED, median (IQR)	30 (15–100)	30 (15–98)	44 (18–129)
≤ 90 mg/day	10,799 (68.7)	10,665 (68.8)	134 (65.4)
>90 mg/day	3,829 (24.4)	3,767 (24.3)	62 (30.2)
Missing MED	1,085 (6.9)	1,076 (6.9)	9 (4.4)
**Types**			
OMT drug	766 (4.9)	759 (4.9)	7 (3.4)
Cough drug	621 (4.0)	614 (4.0)	7 (3.4)
Immediate-release combo opioids	8,414 (53.5)	8,310 (53.6)	104 (50.7)
**Single-agent opioids**			
Codeine	380 (2.4)	374 (2.4)	6 (2.9)
Tramadol	617 (3.9)	4.0%	1.0%
Morphine	958 (6.1)	940 (6.1)	18 (8.8)
Hydromorphone	1,805 (11.5)	1,770 (11.4)	35 (17.1)
Fentanyl	619 (3.9)	608 (3.9)	11 (5.4)
Oxycodone	1,060 (6.7)	1,046 (6.7)	14 (6.8)
Other (e.g., meperidine, pentazocine, buprenorphine)	485 (3.1)	3.1%	1.5%
**Opioid dosage change from index to first refill within 180 days**			
No refill within 180 days	1,407 (9.0)	1,389 (9.0)	18 (8.8)
A dose reduction at first refill	3,896 (24.8)	3,842 (24.8)	54 (26.3)
A dose increase at first refill	1,507 (9.6)	1,491 (9.6)	16 (7.8)
The same dose as at the first refill	7,675 (48.8)	7,569 (48.8)	106 (51.7)
Missing MED	1,228 (7.8)	1,217 (7.8)	11 (5.4)

IQR, interquartile range; MED, morphine equivalent daily dose; OMT, opioid maintenance therapy.

During the first year of follow-up, 205 (1.3%) died from all-cause, 3,077 (19.6%) were hospitalized for all causes, and 6,887 (43.8%) went to ED for all causes. Two hundred and thirteen (1.4%) individuals were hospitalized or went to ED for opioid poisoning.

### The relationship between opioid characteristics and outcomes

#### Primary analysis

**Figure 1:**
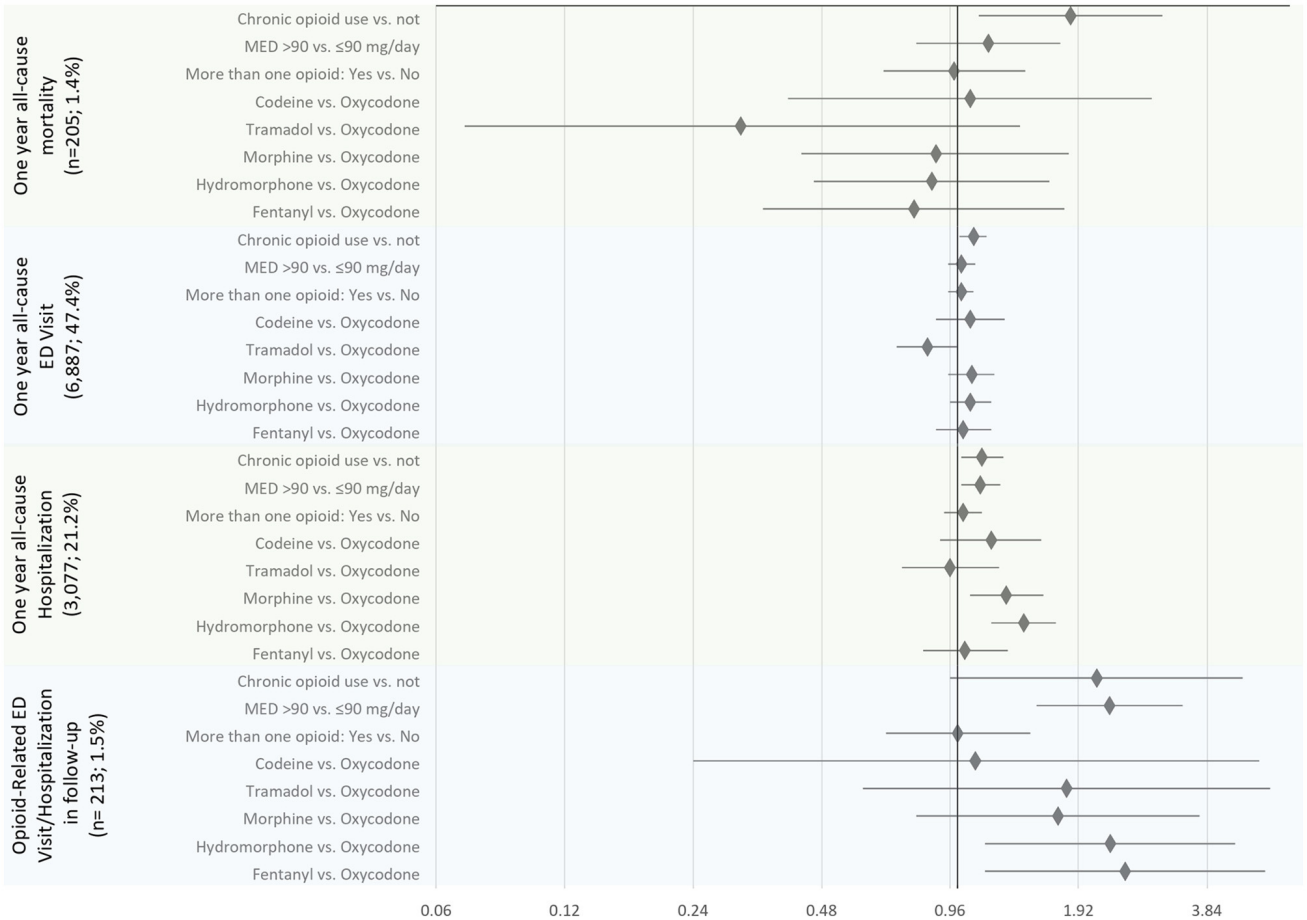
The association between opioid-related characteristics and outcomes of the interest: all-cause mortality, emergency department (ED) visit, and hospitalization within the first year since the diagnostic sleep study, and opioid poisoning-related ED visit and/or hospitalizations (*N* total = 14,532). The estimates are presented as adjusted hazard ratios and 95% confidence intervals.

Of 15,713 individuals, 14,532 (92.5%) were considered for the primary analysis (oral administration of opioids only, excluding those on OMT). The effects of opioid characteristics on outcomes are presented in Table 3 and Figure 1.

**Table 3:** The association between opioid-related characteristics and outcomes of the interest: all-cause mortality, emergency department (ED) visit, and hospitalization within the first year since the diagnostic sleep study, and opioid poisoning-related ED visit and/or hospitalizations (*N* total = 14,532).

**Opioid-related characteristics**	**All-cause mortality**	**All-cause ED visit**	**All-cause hospitalization**	**Opioid poisoning related ED visit and/or hospitalization**
	***N*** = **205**	***N*** = **6,887**	***N*** = **3,077**	***N*** = **213**
	Adjusted^*^ HR (95% Confidence interval)			
**AT THE INDEX DATE (DATE OF THE SLEEP STUDY)**				
**Model 1**				
Chronic opioid use vs. not	**1.84 (1.12–3.02)**	**1.09 (1.01–1.17)**	**1.14 (1.02–1.28)**	2.12 (0.96–4.66)
MED > 90 vs. ≤ 90 mg/day	1.18 (0.80–1.74)	1.02 (0.95–1.10)	**1.13 (1.02–1.26)**	**2.27 (1.53–3.37)**
+1 opioid: yes vs. no	0.98 (0.67–1.44)	1.02 (0.95–1.09)	1.03 (0.93–1.14)	1.00 (0.68–1.48)
**Model 2: comparisons presented for single-agent products, oral administration only, adjusting**				
**additionally for MED and each medication class separately included in the statistical model**.				
Codeine	1.07 (0.40–2.85)	1.07 (0.89–1.29)	1.20 (0.91–1.57)	1.10 (0.24–5.09)
Tramadol	0.31 (0.07–1.40)	0.85 (0.72–1.00)	0.96 (0.74–1.25)	1.80 (0.60–5.40)
Morphine	0.89 (0.43–1.82)	1.08 (0.95–1.22)	**1.30 (1.07–1.59)**	1.72 (0.80–3.69)
Hydromorphone	0.87 (0.46–1.64)	1.07 (0.96–1.20)	**1.43 (1.20–1.70)**	**2.28 (1.16–4.47)**
Fentanyl	0.79 (0.35–1.78)	1.03 (0.89–1.20)	1.04 (0.83–1.31)	**2.47 (1.16–5.26)**
Oxycodone	Reference	Reference	Reference	Reference

The estimates are presented as adjusted hazard ratios and 95% confidence intervals.

In bold: statistically significant associations.

^*^Adjusted for covariates: Age, sex, neighbourhood income status, rurality, alcohol use disorder, separate comorbidities (cardiovascular conditions, COPD, diabetes, hypertension, mental health-related conditions, neuromuscular disease, prevalent cancer), Charlson Comorbidity Index, SBD-related treatment (in follow-up), benzodiazepines dispensed within 1 year prior to the sleep study, any opioid use disorder indication, and the number of primary care visits, having an inpatient surgery, hospitalizations or ED visits 1 year prior to the sleep study.

ED, emergency department; MED, morphine equivalent daily dose.

##### Primary outcome

Controlling for covariates, among opioid-related characteristics tested, only chronic opioid use (vs. not) was significantly associated with an increased hazard of all-cause mortality (HR of 1.84, 95% CI: 1.12–3.02) (Table 3; Figure 1). Among other variables considered in the statistical model, older age, increase in Charlton Comorbidity Index, prior benzodiazepines dispense, alcohol dependence/intoxication, severe COPD, and cancer were also associated with an increased hazard of all-cause mortality (Supplementary Table 2).

##### Secondary outcomes

Chronic opioid use (vs. not; HR: 1.14, 95% CI: 1.02–1.28), a higher dosage of opioids (MED >90 vs. ≤ 90 mg/day; HR: 1.13; 95% CI: 1.02–1.26), morphine or hydromorphone prescription (vs. oxycodone; HR: 1.30, 95% CI: 1.07–1.59 and HR: 1.43, 95% CI, 1.20–1.70, respectively) were significantly associated with an increased hazard of all-cause hospitalizations (Table 3; Figure 1). Chronic opioid use (vs. not) was associated with an increased hazard of all-cause ED visits (HR: 1.09, 95% CI: 1.01–1.17). A higher dosage of opioids (MED >90 vs. ≤ 90 mg/day; HR: 2.27; 95% CI: 1.53–3.37) and hydromorphone or fentanyl prescription (vs. oxycodone; HR: 2.28, 95% CI: 1.16–4.47 and HR: 2.47, 95% CI: 1.16–5.26, respectively) were significantly associated with an increased hazard of opioid poisoning related ED visit and/or hospitalization.

##### Secondary and explanatory analyses

A prescription for benzodiazepines in the year prior to the sleep study did not significantly modify the effect of chronic opioid use on outcomes (all *p*-values for the interaction term > 0.10) (Supplementary Table 3). The effect of MED on outcomes (MED >90 vs. ≤ 90 mg/day) on all-cause mortality was greater among those who were not prescribed benzodiazepines vs. prescribed (HR of 1.95, 95%CI: 1.18–3.23, vs. HR of 0.64, 95% CI: 0.36–1.14; *p*-value for the interaction of < 0.01); other interactions terms were not significant (*p*-values > 0.15) (Supplementary Table 3).

Being on the OMT-relevant therapy was not associated with the outcomes of interest, controlling for confounders (Supplementary Table 4).

No refill within 180 days since the index date (vs. doses remained the same) was significantly associated with a decreased hazard of all-cause mortality, hospitalizations, and opioid poisoning-related ED visit and/or hospitalizations. Inconsistent results were noticed for changes in opioid dose within 180 days since the index date. Changes in dose were not significantly associated with all-cause mortality and opioid poisoning-related ED visit and/or hospitalizations (Supplementary Table 4). Both, an increase or reduction in opioid dose at the first refill were associated with an increased hazard of all-cause ED visits or hospitalizations.

## Discussion

**Figure 2:**
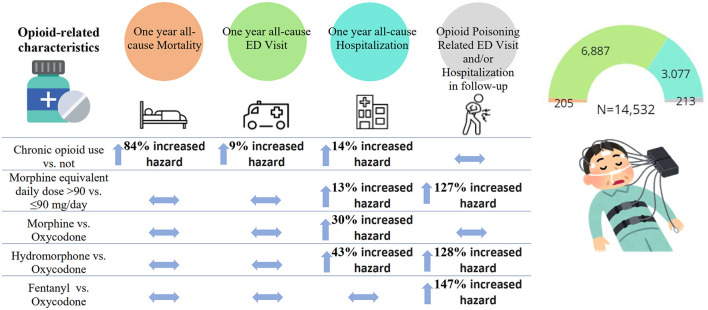
The main study findings. ED, emergency department.

In this retrospective study of ~15,000 adult opioid recipients who underwent a diagnostic sleep study, we identified opioid characteristics associated with a higher hazard of long-term adverse health outcomes. Among different opioid-related characteristics tested, only chronic opioid use was significantly associated with an increased hazard of all-cause mortality and ED visits (Figure 2). Chronic opioid use, a higher dosage of opioids and morphine or hydromorphone prescription (vs. oxycodone) were significantly associated with an increased hazard of all-cause hospitalizations. A higher dosage of opioids and hydromorphone or fentanyl prescription (vs. oxycodone) were significantly associated with an increased hazard of opioid poisoning-related ED visit and/or hospitalization. Given that data on the association between specific characteristics of opioid use and long-term outcomes is limited, our findings may inform the safe opioid prescribing practice in this population to decrease adverse health outcomes.

The current literature supports our findings of a higher hazard of all-cause mortality associated with chronic opioid use. Studies demonstrated increased all-cause mortality associated with selected opioids compared to non-opioid medications for chronic non-cancer pain in the specific population (Ray et al., 2016; Hauser et al., 2020).

In a cohort of individuals with COPD on long-term oxygen therapy, there was no increase in mortality when opioids were started at a MED ≤ 30 mg, regardless of whether an individual was naive to opioids or concurrently used benzodiazepines (Ekstrom et al., 2014). A higher MED > 30 mg was associated with increased mortality (Ekstrom et al., 2014). We did not find a significant effect associated with higher opioid dosage; however, the 95% upper confidence level of 74% increased hazard associated with the MED >90 vs. ≤ 90 mg/day may have an important implication. Nevertheless, all-cause hospitalization and opioid poisoning-related ED visit and/or hospitalization were significantly associated with MED > 90 mg/day in our study.

While opioids may significantly differ in their pharmacokinetics and pharmacodynamics, the outcome of clinical trials comparing one opioid with another may not reach significant differences in either efficacy or safety (Drewes et al., 2013). For example, a systematic review of 15 randomized controlled trials (RCT) on long-term use of strong opioids in individuals with chronic non-cancer patients (Kalso et al., 2004) demonstrated a large individual variation in opioid responses that may explain why no differences were found between opioids in terms of efficacy and safety. However, long-term adverse health outcomes were not considered in those studies.

An increased hazard of opioid poisoning-related ED visit and/or hospitalization associated with fentanyl vs. oxycodone use found in our study may be explained by a higher chance of overdose fentanyl can cause in individuals who are not fully tolerant to opioids (Kahan et al., 2011b). Therefore, fentanyl has been recommended for individuals who have taken an opioid at least 60- to 100-mg MED, for at least 2 weeks (Kahan et al., 2011b). In line with our findings on an increased hazard of all-cause hospitalizations associated with being on morphine or hydromorphone (vs. oxycodone) with a potentially greater hazard associated with hydromorphone, 30-day all-cause readmission rates were found to be significantly higher in individuals treated with hydromorphone compared to morphine (Gulur et al., 2015).

Although our findings on the benefits of discontinuation of opioid therapy within 6 months may be affected by a survival bias, our results support previous findings that long-term opioid therapy (≥6 months) in chronic non-cancer pain may not be superior to non-opioids in improving pain, pain-related function or disability, but seems to be associated with more adverse outcomes and possibly an increase in all-cause mortality (Nury et al., 2022). Thus, avoiding long-term opioids for chronic pain has been suggested (Botticelli et al., 2019). Other considerations include shared decision-making and focus on risk reduction; involuntary and abrupt opioid tapers were suggested as inappropriate (Botticelli et al., 2019). This may explain why we have not demonstrated a beneficial effect of opioid dose reduction at the first refill within 180 days since the index date; on the contrary, we have found an increased hazard of all-cause ED visits and hospitalizations within the first year associated with the dose reduction.

Similar to some published studies, our findings are inconsistent with concerns that concurrent benzodiazepine and opioids use increases the risk of adverse outcomes compared to each medication alone. For example, while similar to our study, treatment with benzodiazepines has been shown to be associated with increased mortality in individuals with COPD, there was no evidence that the effects of benzodiazepines and opioids on mortality were modified by concurrent use (Ekstrom et al., 2014). Conversely, concurrent treatment with benzodiazepines and opioids was associated with a lower admission rate (Ekstrom et al., 2014). However, the authors suggested that results were influenced by performance status and might not reflect a clinically significant synergy (Ekstrom et al., 2014). Further, the concurrent benzodiazepine and opioid use decreased sleep apnea risk in individuals with chronic pain (Mir et al., 2020). Authors hypothesized that in individuals with chronic pain on opioids, administration of certain benzodiazepines induced a mild respiratory depression, but paradoxically reduced sleep apnea risk and severity by increasing the respiratory arousal threshold, potentially stabilizing breathing (Mir et al., 2020).

The strengths of our study are its real-world relevance, its inclusion of a large, complete population of people presumably at risk for opioid complications and its focus on the long-term safety of different opioids and non-opioid-related outcomes. Our study has several limitations. A new-user design for observational effectiveness and drug safety research is recommended to reduce the risk of selection bias in exposure effect estimation compared to a prevalent-user design. However, given the focus of our study on the individuals who underwent a sleep study, we were not able to implement it. Further, it is important to consider confounding by indication. We have tried to minimize the bias by adjusting for prior ED visits, hospitalizations, and surgical interventions. Next, unmeasurable variables could confound the study results. For example, clinical indications for the opioid prescription and results of the sleep study to identify a specific sleep disorder were not available. However, we showed previously a significantly increased hazard of all-cause mortality, hospitalizations and ED visits associated with opioid prescription vs. not, regardless of SDB presence (Kendzerska et al., 2022). Importantly, we demonstrated a higher prevalence of opioid use with a large proportion on long-acting opioids, higher opioid dosages, and on benzodiazepines among adults referred for a sleep disorder assessment than the general population (Kendzerska et al., 2020), suggesting that these individuals are more susceptible to opioid-related adverse health consequences regardless of clinical indications for the opioid prescription or sleep disorder assessment. Finally, the explanatory analysis results are needed to be interpreted with caution, given a potential indication bias and survival bias. For example, opioid discontinuation looks highly protective against all-cause mortality, but that is heavily confounded by indication since when an individual dies, it gives them less time to accrue the requisite follow-up time to discontinue the drugs. Future studies are also required to understand the modifiable effect of severity and nature of sleep disorders on safe opioid prescription, even in those who were not referred for diagnostic sleep assessments.

## Conclusions

In this health administrative data study on adult opioid recipients who underwent a diagnostic sleep study, we identified opioid characteristics associated with a higher hazard of adverse health outcomes. Chronic opioid use (vs. not) was significantly associated with increased hazards of all-cause mortality, hospitalizations or ED visits. A higher opioid dosage was significantly associated with increased hazards of all-cause or opioid-related hospitalizations. Morphine or hydromorphone prescription (vs. oxycodone) was significantly associated with an increased hazard of all-cause hospitalizations. Hydromorphone or fentanyl prescription (vs. oxycodone) was significantly associated with an increased hazard of opioid-related ED visit and/or hospitalization. If confirmed in future studies, these findings may inform the safe prescribing of opioids in this population.

## Data availability statement

The datasets presented in this article are not readily available because the dataset from this study is held securely in coded form at ICES. While data sharing agreements between ICES and data providers (e.g., healthcare organizations and government) prohibit ICES from making the dataset publicly available, access may be granted to those who meet pre-specified criteria for confidential access, available at www.ices.on.ca/DAS (email: das@ices.on.ca). The full dataset creation plan and underlying analytic code are available from the authors upon request, understanding that the computer programs may rely upon coding templates or macros that are unique to ICES and are, therefore, either inaccessible or may require modification. Requests to access the datasets should be directed to www.ices.on.ca/DAS (das@ices.on.ca).

## Ethics statement

Ethical review and approval was not required for the study on human participants in accordance with the local legislation and institutional requirements. Written informed consent for participation was not required for this study in accordance with the national legislation and the institutional requirements. ICES is a prescribed entity under Ontario's Personal Health Information Protection Act (PHIPA). Section 45 of PHIPA authorizes ICES to collect personal health information, without consent, for the purpose of analysis or compiling statistical information with respect to the management of, evaluation or monitoring of the allocation of resources to or planning for all or part of the health system. Projects that use data collected by ICES under section 45 of PHIPA, and use no other data, are exempt from REB review. The use of the data in this project is authorized under section 45 and approved by ICES' Privacy and Legal Office.

## Author contributions

TK was involved in the literature search, data analyses, and manuscript drafting. RT was involved in data analyses. TK and RT had full access to all the data in the study and took responsibility for the integrity of the data and accuracy of the data analysis. All authors were involved in the study conception and design, interpretation of data, revising the manuscript critically for the accuracy and important intellectual content, and final approval of the version to be published.
