# Supplementary material for: Association between opioid prescription profiles and adverse health outcomes in opioid users referred for sleep disorder assessment: a secondary analysis of health administrative data
Source: Front Sleep. 2023 Jul 7;2:1161857. doi: 10.3389/frsle.2023.1161857 (PMC12713942; doi:10.3389/frsle.2023.1161857)
Supplement: Supplementary file 1 [file Data_Sheet_1.pdf]

Data Supplement

Title: Association between Opioid Prescription Profiles and Adverse Health Outcomes in Opioid Users Referred for Sleep Disorder Assessment: A Secondary Analysis of Health Administrative Data

Authors: Tetyana Kendzerska, Atul Malhotra, Andrea S. Gershon, Marcus Povitz, Daniel I. McIsaac, Shawn D. Aaron, Gregory L. Bryson, Robert Talarico, Michael Godbout, Peter Tanuseputro, Frances Chung

Text E1: Details on ICES databases used.

Tables
Table E1: Details on the cohort creation and variable definitions.
Table E2: The results of the multivariable Cox regression analysis on the association between opioid-related characteristics and the primary outcome, all-cause mortality within the first year since the diagnostic sleep study, in adults who underwent a diagnostic sleep study between 2013 and 2016 while being treated with prescription opioids. Estimates are presented as adjusted hazard ratios and 95% confidence intervals.
Table E3: The effect of the prescription of benzodiazepines in the last year prior to the sleep study on the relationship between opioid characteristics and the outcome of the interest.
Table E4: The association between opioid maintenance therapy (OMT) at the date of the sleep study and opioid dosage change at the first refill and opioid discontinuation within 180 days of the date of the sleep study. Estimated presented as adjusted hazard ratios and confidence interval.

E-References

Text E1: Details on ICES databases used.

Since 1991, ICES (www.ices.on.ca) has housed high-quality administrative datasets on publicly funded services provided, including individual-level information on physician claims, hospitalization, and emergency visits within Ontario.[1] The accuracy of these datasets has been previously validated.[2,3] The Registered Persons Database (RPDB) contains data on demographics, residence location, and date of death; the Discharge Abstract Database records up to 25 diagnoses and procedures performed for each hospital admission; the National Ambulatory Care Reporting System Database records up to 10 diagnoses and procedures for emergency room and urgent care visits; the OHIP database captures all physician billing and technical fees for procedures such as PSG, and the Canadian Census includes neighborhood socioeconomic details. The Narcotics Monitoring System (NMS) collects data on dispensed prescriptions for narcotics, controlled substances, and other monitored drugs such as stimulants, benzodiazepines/zolpidem, and barbiturates. Since November 1, 2011, all dispensers in Ontario are required to submit information to the NMS on date, name, strength, dosage of the monitored drug, quantity dispensed, and length of therapy. Furthermore, for all insured Ontario residents who have been diagnosed with SDB by a sleep physician, funding is provided for PAP systems and documented in the Assistive Devices Program (ADP) database from 2000 onwards.[4] There are several positive airway pressure (PAP) modalities used to treat patients with sleep disordered breathing (SDB): CPAP, autotitrating positive airway pressure (APAP), bilevel positive airway pressure (BiPAP) and adaptive servo-ventilation (ASV). The Respiratory Equipment Pool of the ADP provides 75% of the cost of a basic CPAP/APAP/BiPAP device. The Ventilatory Equipment Pool of the ADP provides ventilators including BiPAP required back up rate and is fully funded by the government. A description of the ICES datasets is available at https://datadictionary.ices.on.ca/Applications/DataDictionary/Default.aspx.

Table E1: Details on the cohort creation and variable definitions.

**<u>Cohort of Interest</u>: individuals who underwent diagnostic sleep study, untreated for sleep disordered breathing, with active opioid prescription at the sleep study (the index date)**	**Cohort**: All adults (***18+***) who underwent a diagnostic sleep study (***index study date***) identified using the OHIP fee codes (J890, J690, J896, J696, J897, J697) <u>from July 2013 and June 2016</u> AND with active opioid prescription at the index date (please see details below)<br>**Excluded individuals**:<br>(1) received ***palliative care*** (based on physician service codes in OHIP and CIHI-DAD databases) <u>in the year prior to the index date</u>: admitted to hospital with a patient service code for palliative care (PATSERV = 58) or palliative diagnostic code (Z515) in any diagnosis field; or if a treating physician had billed OHIP for any of the following palliative care fee codes: A945, B998, C945, C882, C982, K023, W872, W882, W972 or W982; or palliative end of life homecare (service code 95 or 54) from home care delivered services OR<br>(2) in ***long-term care*** (LTS) <u>in the year prior to the index date</u> from Continuing Care Reporting System (CCRS - LTC) OR<br>(3) already on ***positive airway pressure (PAP) treatment*** at the <u>index date</u> or requested a repeat PAP prescription <u>in the last 5 years</u> through the Assistive Device Program Database (ADP) OR<br>(4) underwent a ***therapeutic sleep study*** (the OHIP fee codes: J889, J689, J895, J695) <u>in the last 5 years</u> preceding the index date OR<br>(5) were taking opioids that are rarely used and/or with no well-defined morphine equivalencies such as intranasal, injectable, or rectal suppositories opioids at the index date OR<br>(6) Missing age or gender OR<br>(7) Uninsured
**Opioids of interest** (the Narcotic Monitoring System [NMS] database)	Individuals on opioids will be defined through the NMS database by dispensing of **oral/transdermal** opioids between July 2012 and March 2018.<br>At least one opioid prescription over the study period, including **oral formulations** of morphine, codeine, oxycodone, meperidine, hydromorphone, pentazocine, tramadol, tapentadol, opium (miscellaneous opioids) as well as **transdermal** fentanyl and buprenorphine patches, and opioid maintenance therapy (OMT), that includes buprenorphine for opioid dependence (Subutex), buprenorphine/naloxone, and methadone for opioid dependence.
**Active opioid prescription**	An opioid Rx that overlaps with the index date (i.e., date of dispensing is < index date and date of dispensing + days supply is >index date)
**Exposures: Opioid characteristics at the index date**	
**Chronic opioid use**	Three or more prescriptions for any opioids in the last six months or at least one prescription for a long-acting opioid[5]
**The daily opioid dose (morphine equivalent daily dose, MED)**	• The daily dose was calculated as the total dose (in milligrams) divided by the number of days' supply for which the prescription was written, converted to morphine equivalents using morphine equivalence ratios used by the Canadian National Opioid Use Guideline Group. When multiple concurrent opioid prescriptions were identified, the total average daily dose was defined as the sum of the average daily dose of all prescriptions overlapping the patient's index date.<br>• MED was considered as both a continuous variable and categorical variable (<90 vs. ≥90 mg/day)[6,7]
**Outcomes**	

**Withing the first year since the index date**	
**Primary outcome: All-cause mortality (from RPDB – Demographic database)**	The date of the death from all-causes
**Secondary outcomes**	
**All-cause Hospitalization (from DAD database)**	The date of inpatient hospitalization for all-causes
**All-cause Emergency Department Visit that does not result in a hospitalization (from NACRS database)**	The date of emergency department visit for all-causes
**From the index date to the last date of the follow-up** (***March 31, 2018***)*	
**Opioid Poisoning Related ED Visit and/or Hospitalization** [8,9]	The date of hospitalizations and/or ED visits for (ICD-10-CA codes) [10]:<br>– T40.0 (poisoning by opium)<br>– T40.1 (poisoning by heroin)<br>– T40.2 (poisoning by other opioids)<br>– T40.3 (poisoning by methadone)<br>– T40.4 (poisoning by other synthetic narcotics)<br>– T40.6 (poisoning by other and unspecified narcotics)
**Variable Definitions (Baseline Characteristics)**	
**Baseline demographics**	– Age, sex<br>– Socioeconomic status (SES): A patient's residential neighbourhood income was defined from the Ontario Census. Ontario neighbourhoods are classified into one of the five approximately equal-sized income quintiles, ranked from poorest (Q1) to wealthiest (Q5) and shown to be related to population health status and health care utilization.[11] The neighborhood income quintiles have previously been shown to be a useful method to stratify individuals by SES and to identify related disparities in health and health care utilization.[11] Research has demonstrated that the neighborhood-level income measures may not only account for the aspects of individual-level SES, such as income and education level, but also measure contextual factors of SES, such as access to resources, availability and quality of local services, rates of crime and violence, unemployment rates, and features of the social environment (e.g., social interaction, physical activity).[12,13]<br>– Location of residence (urban vs. rural)
**Information on all medications available in NMS database <u>one year prior to the index date</u>**	– being on any opioids prescribed in past year (Yes/No)<br>– being on benzodiazepines (Yes/No)<br>– being on barbiturates (Yes/No)<br>– being on cannabinoids (Yes/No)<br>– being on stimulants (Yes/No)<br>– being on testosterone (Yes/No)
**Opioid use disorder**	
**Individuals who were hospitalized for opioid use disorder in the last five years preceding the index date (from CIHI)**	Hospitalizations and/or ED visits for (ICD-10-CA codes):<br>• T40.0 (poisoning by opium)<br>• T40.1 (poisoning by heroin)<br>• T40.2 (poisoning by other opioids)<br>• T40.3 (poisoning by methadone)<br>• T40.4 (poisoning by other synthetic narcotics)

	• T40.6 (poisoning by other and unspecified narcotics)[10]
**Individuals who were taking narcotics for opioid use disorder in the last year prior the index date (from NMS database)**	Anyone taking orally methadone and buprenorphine (including in combination with naloxone, e.g., Suboxone)
**Mental and behavioural disorders due to use of opioids (from DAD, NACRS and OMHRS databases) in the last 5 years**	(1) from DAD and NACRS (hospitalizations/ED visits):<br>• ICD-10: F11 (Mental and behavioural disorders due to use of opioids. Details are in the Appendix)<br>(2) from OMHRS:<br>• 304.00: Opioid dependence<br>• 305.50: Opioid abuse
**Prior comorbidities: Comorbidities at index date that can also affect prescription of opioids: hypertension, diabetes, depression, psychiatric comorbidities, liver disease, asthma, COPD (including severe COPD which may require an opioid prescription), cardiovascular disorders, chronic renal disorder, prior health care utilization and surgical interventions; comorbidities associated with early mortality (e.g., cancer and being on dialysis); conditions that can contribute to sleep disordered breathing such as neuromuscular disorders and alcohol intoxication/abuse.**	
**17 ICES chronic conditions at the index date (https://datadictionary.ices.on.ca/Applications/DataDictionary/Default.aspx)**	Validated algorithms[14-22] were used to ascertain cases of the following 8 chronic conditions:<br>1. Acute myocardial infarction[20]<br>2. Asthma[16]<br>3. Congestive Heart Failure[14]<br>4. COPD (sensitive cohort)[15]<br>5. Dementia[17]<br>6. Diabetes (the Ontario Diabetes Database)[18]<br>7. Hypertension (the Hypertension Database)[22]<br>8. Rheumatoid Arthritis (the Ontario Rheumatoid Arthritis Database)[21]<br><br>The remaining 10 chronic conditions were defined according to inpatient hospital diagnostic codes (at least 1 from DAD) or outpatient physician billing codes (at least 2 from OHIP within a 2-year period):<br>1. Arrhythmia<br>2. Coronary Heart Disease<br>3. IBD<br>4. Non-psychotic mood and anxiety disorders<br>5. Osteoarthritis<br>6. Osteoporosis<br>7. Other mental health conditions<br>8. Stroke
**Measure of Comorbidity**	The Charlson comorbidity index (CCI)[23] 2 years prior to index, aggregated, n (%):<br>• none (CCI score = 0)<br>• low (score = 1)<br>• moderate (score = 2)<br>• high (score ≥ 3)
**Presumably moderate to severe COPD**	Prevalent COPD from the ICES-derived COPD specific cohort[15]
**Hospitalizations with serious liver disease** in the	from DAD/SDS:<br>– ICD-9: 5712, 5715, 5716<br>– ICD-10: K703, K71.7, K74

last 5 years prior the index date	
**End stage renal disease, hemodialysis** in the last 5 years prior the index date (from DAD, SDS, NACRS, and/or OHIP databases)	• Any hospitalization or same day record from DAD, or NACRS<br>– ICD-9: 4031, 4039, 585, V45.1<br>– ICD-10: I12, I13, N18.3, 18.4, 18.5, 18.6, 18.9, E08.22, E09.22, E10.22, E11.22, E13.22, Z99.2<br>• OHIP codes: G860, G861, G862, G863, G864, G865, G866
**Cancer**	Prevalent cancer from the Ontario Cancer Registry[24]
**Number of the office visit, primary care**, in the last year prior the index date	Obtain all OHIP records for the desired period, where visit location was in the physician office, LTC (the physician came to a long-term care facility to see the patient), or home (i.e., patient's home)
**Any outpatient or inpatient surgical intervention in the last year**	From DAD database, using intervention indicator
**In the last 5 years**	
**Alcohol dependence/ intoxication** (from DAD, SDS, NACRS, and/or OHIP databases)	• Any hospitalization, ED visit or same day record from DAD, SDS or NACRS<br>– ICD-9: 303, 3050<br>– ICD-10: E512, F10, G312, G621, G721, I426, K292, K70, K860, T510, X45, X65, Y15, Y573, Z502, Z714, Z721<br>• OHIP code: 303
**Neuromuscular Disease** [25] (from DAD, OHIP databases)	– ICD-9, ICD-10 and OHIP codes for the following conditions: Amyotrophic lateral sclerosis, Cerebral palsy, Guillain-Barre syndrome, Metabolic disorders, Multiple sclerosis, Muscular dystrophy, Myasthenia gravis, Neuromuscular disorders (other), Neuropathy, Post-polio syndrome, Spina bifida, Spinal muscular atrophy<br>– For patients identified with OHIP dx349, including only those with subsequent or previous NMD-related ED, hospitalization visit or with subsequent or previous neurologist visit and EMG
**Sleep disordered breathing (SDB)-related treatment** (positive airway pressure therapy or surgical interventions) **in follow-up**	
**PAP treatment initiation (from ADP. Respiratory and ADP. Ventilatory)**	Application for Funding Respiratory Equipment & Supplies forms from which data was extracted is available through the ADP website: https://www.ontario.ca/page/respiratory-equipment-and-supplies. Individuals were defined through the ADP Respiratory and Ventilator Equipment Pool as one who received a government funded CPAP, APAP, and bilevel therapy. The date of the PAP claim will be considered as the date of PAP initiation.
**Bariatric surgery (from CIHI/DAD/ SDS, procedures; OHIP)**	In-patient Bariatric Procedures:<br>• ICD-10-CA – E66, obesity (all codes in category); AND<br>• the Canadian Classification of Health Interventions (CCI) codes: 1NF78 repair by decreasing size, stomach<br>Outpatient bariatric procedures (OHIP FEECODES):<br>• S120 for gastric bypass with Roux-en-Y anastomosis<br>• S114 for sleeve gastrectomy<br>• S189 for intestines-intestinal bypass for morbid obesity
**Maxillomandibular advancement (MMA)/ uvulopalatopharyngoplasty (UPPP) (from CIHI/DAD/ SDS, procedures)**	CCI codes:<br>• MMA: 1EE79<br>• UPPP: 1FQ78LA

ADP, Assistive Devices Program Database; DAD, Discharge Abstract Database (Canadian Institute for Health Information); NACRS, National Ambulatory Care Reporting System Metadata (Canadian Institute for Health Information); CCI, Canadian Classification of Health Interventions; COPD, chronic obstructive pulmonary disease; ED, emergency department; ICD, International Classification of Diseases; NMS, Narcotics Monitoring System; OHIP, the Ontario Health Insurance Plan Database; OMHRS, the Ontario Mental Health Reporting System; PAP, positive airway pressure; RPDB, the Registered Persons Database; SDS, Same Day Surgery; SDB, sleep disordered breathing.

*Given a small sample size for the opioid-related outcome within the first year since the index date, we extended the follow-up for this outcome until the end of the study (March 31, 2018) to increase statistical power.

Table E2: The results of the multivariable Cox regression analysis on the association between opioid-related characteristics and the primary outcome, all-cause mortality within the first year since the diagnostic sleep study, in adults who underwent a diagnostic sleep study between 2013 and 2016 while being treated with prescription opioids. Estimates are presented as adjusted hazard ratios and 95% confidence intervals.

Variables considered in the statistical model		All-cause Mortality within the first year since the diagnostic sleep study<br>Hazard Ratio (95% CI)
**Opioid-related characteristics**		
Chronic opioid use vs. not		**1.84 (1.12-3.02)**
Being on more than one opioid at the index date vs. not		0.98 (0.67-1.44)
MED >90 vs. ≤90 mg/day		1.18 (0.80-1.74)
Demographics at the index date [the date of the diagnostic sleep study]		
Age, years, per one year increase		**1.04 (1.03-1.06)**
Sex: Female vs. Male		0.89 (0.67-1.20)
Neighbourhood Income Quintile (Q)	Q2 vs. Q1 (lowest)	0.75 (0.51-1.10)
	Q3 vs. Q1 (lowest)	0.77 (0.52-1.16)
	Q4 vs. Q1 (lowest)	0.58 (0.36-0.94)
	Q5 (highest) vs. Q1 (lowest)	0.83 (0.52-1.32)
Rurality: No vs Yes		0.78 (0.55-1.12)
Comorbidities, primary health care exposure, surgical interventions and controlled substance use in the last year prior to the sleep study		
Charlson Comorbidity Index (CCI)	High (CCI =3) vs. None (CCI = 0)	**3.67 (2.30-5.86)**
	Moderate (CCI =2) vs. None (CCI = 0)	**2.32 (1.38-3.89)**
	Low (CCI =1) vs. None (CCI = 0)	**2.00 (1.24-3.21)**
Number of Primary Care Visits, per unit increase		1.00 (0.99-1.02)
Surgery/Intervention Indicator: Yes vs. No		0.74 (0.47-1.17)
Number of Hospitalization/ED Visits, per unit increase		1.01 (0.97-1.06)
Benzodiazepines Dispensed		**1.10 (0.82-1.48)**
Comorbidities defined in the last five years prior to the sleep study: Yes vs. No		
Alcohol Dependence/intoxication		**1.73 (1.05-2.83)**
Neuromuscular Disease		1.40 (0.94-2.08)
Prevalent comorbidities: Yes vs. No		
Arrythmia		1.20 (0.82-1.76)
Chronic heart failure		1.46 (1.00-2.13)
Chronic obstructive pulmonary disease		**2.16 (1.58-2.96)**
Coronary artery disease		0.94 (0.67-1.31)
Diabetes		1.04 (0.75-1.43)
Hypertension		1.13 (0.77-1.66)
Non-psychotic Mood and Anxiety Disorders prevalent		0.95 (0.69-1.30)
Cancer		**1.53 (1.06-2.21)**
Prior opioid use disorder		
Any Opioid Use Disorder Indication*: Yes vs. No		1.01 (0.55-1.87)
Follow-up-related variables		
**OSA-relevant treatment in follow-up**		0.86 (0.61-1.23)

In bold: statistically significant associations.

* Any opioid use disorder indication: hospitalizations/ED visits for opioid use disorder or mental and behavioural disorders due to the use of opioids 5-years prior to the index date or taking narcotics for opioid use disorder 1-year prior to the index date.

CCI, Charlson Comorbidity Index; ED, emergency department; MED, morphine equivalent daily dose.

Table E3: The effect of the prescription of benzodiazepines in the last year prior to the sleep study on the relationship between opioid characteristics and the outcome of the interest.

**Description**	All-cause Mortality-1yr	P-value*	All-cause ED Visit1-yr	P-value*	All-cause Hospitalization-1yr	P-value*	Opioid Poisoning Related ED Visit and/or Hospitalization	P-value*
**The effect of chronic opioid use on outcomes**								
Prescription of benzodiazepines in the last year: No	1.86 (0.99-3.48)	0.8842	1.08 (0.99-1.18)	0.9579	**1.21 (1.06-1.39)**	0.1665	2.69 (0.81-8.96)	0.5107
Prescription of benzodiazepines in the last year: Yes	1.72 (0.77-3.84)		1.08 (0.95-1.23)		1.03 (0.85-1.25)		1.58 (0.56-4.45)	
**The effect of morphine equivalent daily dose (MED) on outcomes**								
Prescription of benzodiazepines in the last year: No	**1.95 (1.18-3.23)**	0.0043	1.01 (0.91-1.11)	0.6136	1.10 (0.96-1.27)	0.5609	**3.34 (1.71-6.52)**	0.1591
Prescription of benzodiazepines in the last year: Yes	0.64 (0.36-1.14)		1.05 (0.94-1.16)		**1.17 (1.01-1.36)**		**1.85 (1.14-3.01)**	

*p-values for the statistical interaction term

ED, emergency department

Table E4: The association between opioid maintenance therapy (OMT) at the date of the sleep study and opioid dosage change at the first refill and opioid discontinuation within 180 days of the date of the sleep study. Estimated presented as adjusted hazard ratios and confidence interval.

**Opioid-related characteristics**	**All-cause Mortality-1yr**	**All-cause ED Visit-1yr**	**All-cause Hospitalization-1yr**	**Opioid Poisoning Related ED Visit and/or Hospitalization**
	N= 205	N= 6,887	N= 3,077	N=213
**At the index date (date of the sleep study), N = 15,678**				
OMT: Yes vs. No	1.22 (0.43-3.42)	0.85 (0.72-1.00)	0.80 (0.61-1.04)	1.55 (0.93-2.57)
**Changes in dose within 180 days since the index date, N = 14,532**				
No refill within 180 days: Yes vs. No	**0.06 (0.02-0.18)**	0.95 (0.89-1.01)	**0.88 (0.80-0.96)**	**0.48 (0.31-0.77)**
Dose reduction at the first refill	1.16 (0.83-1.61)	**1.11 (1.04-1.17)**	**1.18 (1.08-1.28)**	1.38 (0.99-1.91)
Dose increase at the first refill	0.89 (0.52-1.50)	**1.24 (1.13-1.35)**	**1.26 (1.11-1.43)**	0.69 (0.37-1.30)
The same dose at the first refill	Reference	Reference	Reference	Reference

In bold: statistically significant associations.

ED, emergency department; OMT, opioid maintenance therapy

E-References:

1. Improving health care data in Ontario. *ICES investigative report Toronto: Institute for Clinical Evaluative Sciences.* 2005.
2. Juurlink D PC, Croxford R, Chong A, Austin P, Tu J, Laupacis A. . *Canadian Institute for Health Information Discharge Abstract Database: A Validation Study* Toronto: Institute for Clinical Evaluative Sciences;2006.
3. Goel V, Canadian Medical Association., Institute for Clinical Evaluative Sciences in Ontario. *Patterns of health care in Ontario.* 2nd ed. Ottawa: Published by the Canadian Medical Association [for] the Institute for Clinical Evaluative Sciences in Ontario; 1996.
4. Assistive Devices Program. Continuous/Autotitrating/BiLevel Positive Pressure Systems. Accessed July 30, 2016, 2016.
5. Kendzerska T, Gomes T, Gershon AS, et al. Opioid use and initiation of positive airway pressure treatment in adults referred for sleep disorder assessment: An explanatory population-based study. *Canadian Journal of Respiratory, Critical Care, and Sleep Medicine* 2020;4(3):194-204.
6. Busse JW, Craigie S, Juurlink DN, et al. Guideline for opioid therapy and chronic noncancer pain. *CMAJ : Canadian Medical Association journal = journal de l'Association medicale canadienne.* 2017;189(18):E659-E666.
7. Kurteva S, Abrahamowicz M, Gomes T, Tamblyn R. Association of Opioid Consumption Profiles After Hospitalization With Risk of Adverse Health Care Events. *JAMA Netw Open.* 2021;4(5):e218782.
8. Fernandes K, Martins D, Juurlink D, et al. High-Dose Opioid Prescribing and Opioid-Related Hospitalization: A Population-Based Study. *PloS one.* 2016;11(12):e0167479.
9. Understanding a broader range of opioid-related hospitalizations in Canada. Goverment of Canada. https://www.canada.ca/en/health-canada/services/opioids/data-surveillance-research/understanding-broader-range-opioid-related-hospitalizations.html. Published 2021. Accessed Sep 4, 2022.
10. *Quality Standards. Opioid Use Disorder Measurement Guide.* 2018.
11. Wilkins R, Tjepkema M, Mustard C, Choiniere R. The Canadian census mortality follow-up study, 1991 through 2001. *Health reports / Statistics Canada, Canadian Centre for Health Information = Rapports sur la sante / Statistique Canada, Centre canadien d'information sur la sante.* 2008;19(3):25-43.
12. Pampalon R, Hamel D, Gamache P. A comparison of individual and area-based socio-economic data for monitoring social inequalities in health. *Health reports / Statistics Canada, Canadian Centre for Health Information = Rapports sur la sante / Statistique Canada, Centre canadien d'information sur la sante.* 2009;20(4):85-94.
13. Holowaty EJ, Norwood TA, Wanigaratne S, Abellan JJ, Beale L. Feasibility and utility of mapping disease risk at the neighbourhood level within a Canadian public health unit: an ecological study. *International journal of health geographics.* 2010;9:21.
14. Schultz SE, Rothwell DM, Chen Z, Tu K. Identifying cases of congestive heart failure from administrative data: a validation study using primary care patient records. *Chronic diseases and injuries in Canada.* 2013;33(3):160-166.
15. Gershon AS, Wang C, Guan J, Vasilevska-Ristovska J, Cicutto L, To T. Identifying individuals with physcian diagnosed COPD in health administrative databases. *COPD.* 2009;6(5):388-394.
16. Gershon A WC, Vasilevska-Ristovska J, Guan J, Cicutto L, To T. Identifying patients diagnosed with asthma using health administrative data. *Canadian Respiratory Journal.* 2009;16:183–188.
17. Jaakkimainen RL, Bronskill SE, Tierney MC, et al. Identification of Physician-Diagnosed Alzheimer's Disease and Related Dementias in Population-Based Administrative Data: A

Validation Study Using Family Physicians' Electronic Medical Records. *J Alzheimers Dis.* 2016;54(1):337-349.
18. Hux JE, Ivis F, Flintoft V, Bica A. Diabetes in Ontario: determination of prevalence and incidence using a validated administrative data algorithm. *Diabetes Care.* 2002;25(3):512-516.
19. Tu K, Campbell NR, Chen ZL, Cauch-Dudek KJ, McAlister FA. Accuracy of administrative databases in identifying patients with hypertension. *Open Med.* 2007;1(1):e18-26.
20. Austin PC, Daly PA, Tu JV. A multicenter study of the coding accuracy of hospital discharge administrative data for patients admitted to cardiac care units in Ontario. *American heart journal.* 2002;144(2):290-296.
21. Widdifield J, Bernatsky S, Paterson JM, et al. Accuracy of Canadian health administrative databases in identifying patients with rheumatoid arthritis: a validation study using the medical records of rheumatologists. *Arthritis care & research.* 2013;65(10):1582-1591.
22. Public Health Agency of Canada. Report from the Canadian Chronic Disease Surveillance System hypertension in Canada, 2010. In: [Ottawa]: Public Health Agency of Canada; 2010: http://myaccess.library.utoronto.ca/login?url=http://site.ebrary.com/lib/utoronto/Top?id=10425643

http://myaccess.library.utoronto.ca/login?url=http://site.ebrary.com/lib/utoronto/Top?id=10425670.
23. Sundararajan V, Henderson T, Perry C, Muggivan A, Quan H, Ghali WA. New ICD-10 version of the Charlson comorbidity index predicted in-hospital mortality. *Journal of clinical epidemiology.* 2004;57(12):1288-1294.
24. McLaughlin JR, Kreiger N, Marrett LD, Holowaty EJ. Cancer incidence registration and trends in Ontario. *Eur J Cancer.* 1991;27(11):1520-1524.
25. Rose L, McKim D, Leasa D, et al. Trends in incidence, prevalence, and mortality of neuromuscular disease in Ontario, Canada: A population-based retrospective cohort study (2003-2014). *PLoS One.* 2019;14(3):e0210574.
